# Comparative outcomes for pediatric cranial procedures by general surgeons and neurosurgeons in a tertiary care hospital in Ethiopia

**DOI:** 10.1007/s00381-025-06979-y

**Published:** 2025-10-27

**Authors:** Cleresa Renee Roberts, Oumou Kalsoum Mbacke, Divine Nwafor, Emily Dunbar, Abhinav Kareddy, Maya Parker, Joy Pierson, Bereket Hailu Mekuria, Heather Stevens Spader

**Affiliations:** 1 University of Virginia Medical Center, 204 W Main St 5th Floor, Charlottesville, VA 22903 USA; 2 Soddo Christian General Hospital, Wolaita Sodo, Ethiopia

**Keywords:** Task sharing, Global neurosurgery, Ethiopia, Sub-Saharan Africa, Traumatic brain injury, Pediatric neurosurgery

## Abstract

**Purpose:**

In resource-limited hospitals, pediatric neurosurgery is performed by general surgeons (GS) and neurosurgeons (NS), but the provider-specific impact remains unclear. This study compares pediatric neurosurgery cranial cases managed by GS and NS at a regional referral center in Southern Ethiopia.

**Methods:**

Cranial operations in patients < 18 years (January 2020–October 2024) were retrospectively reviewed. Data included demographics, travel, delay (> 12 h), procedure type, and early outcomes (complications, length of stay [LOS]). Procedures were classified as routine (elevation, washout/I&D) or complex (shunt, craniotomy). Chi square with Yates and Mann‑Whitney U tests compared groups. Multivariable logistic regression adjusted for age, sex, and delay.

**Results:**

Among the 75 patients, 36 (48%) were treated by NS and 39 (52%) by GS. Routine operations predominated in GS (69.2% vs 55.6%) while complex procedures were more frequent in NS (36.1% vs 5.1%). LOS was comparable (4 days, p = 0.772), as were delays (41.0% GS vs 44.4% NS, p = 0.818) and complications (12.8% GS vs 19.4% NS, χ2 test with Yates p = 0.641). Complication rates for routine procedures were similar between groups (elevation 0% and washout/I&D 40%; p = 1.000), and although unadjusted rates were higher in the NS cohort for complex cases (craniotomy 66.7% vs 30.0%; shunt 23.1% vs 0%), these differences were not statistically significant (p = 0.510 and p = 1.000, respectively), with multivariable adjustment likewise showing no provider effect (aOR 1.25, 95% CI 0.27–5.78; p = 0.773).

**Conclusions:**

With appropriate training, GS achieved early outcomes comparable to NS for routine and complex pediatric cranial operations. The absence of significant differences in complications supports structured task‑sharing and skills-based mentorship to expand neurosurgical care in resource-limited settings.

## Introduction

Timely pediatric neurosurgical care can avert death and lifelong disability for vast numbers of children worldwide. Yet, a 2017 analysis estimated that 1.7 billion children and adolescents still lack access to any surgical services [1]. Of the roughly 2,297 pediatric neurosurgeons (NS) in practice globally, 85.6% are concentrated in high‑ and upper‑middle‑income nations.^2^ That distribution leaves 330 specialists to serve more than one billion children in low‑ and lower‑middle‑income countries (LMICs). Nowhere is the shortage more pronounced than in Africa, where roughly one pediatric NS is available for every 30 million children [2].

Ethiopia, Africa’s second‑most populous country, illustrates the crisis. In 2025, its population reached 135.5 million, with 59.5 million under 18 years [3, 4]. As of 2024, the nation counted only one fellowship‑trained pediatric NS. The pediatric volume therefore falls largely to general NS [5]. Workforce maldistribution compounds the problem: While 76.8% of Ethiopia’s population resides in rural areas, 69% of neurosurgeons are concentrated in cities—over half (56%) in Addis Ababa—leaving many families to travel two to four hours or more to access specialist care [5, 6].

Given that an estimated 23,000 additional NSs are needed globally [7, 8], task sharing (TS) delegating selected neurosurgical procedures to non‑specialists such as general surgeons (GS) has emerged as a pragmatic strategy for health‑equity. TS models for adult neuro‑trauma are already established in settings such as the Philippines, Australia, and several other LMICs, where rigid protocols and continuous mentorship have produced mortality and complication rates comparable to NS‑led care [8–10].

By contrast, evidence for pediatric TS remains scarce. During the study period, pediatric neurosurgery in Ethiopia was delivered primarily by general NSs rather than fellowship-trained pediatric subspecialists, and coverage outside Addis Ababa was limited. In Ethiopia’s southern region, the regional referral hospital’s sub-region had one NS, and, across the broader southern region, there were 7 total (Hawassa 4; Worabe 2; Soddo 1), leaving peripheral facilities reliant on GSs for emergent pediatric cranial cases. [5, 6].

At the regional referral hospital in Southern Ethiopia, GSs perform most urgent cranial operations in children, yet no study has systematically compared their outcomes with those of NSs. To fill this gap, we retrospectively reviewed five years of pediatric cranial cases examining complication rates, presentation delays, and procedure complexity to determine whether a structured TS framework can safely expand access to pediatric neurosurgery in resource‑limited settings. The findings are intended to guide policy measures, training programs, mentorship structures, and quality-assurance systems required to scale these models nationwide.

## Methods

### Study design

This retrospective cohort study was conducted at a tertiary referral hospital in Ethiopia, which serves as a principal center for neurosurgical and general surgical care. The study period included all patients who underwent surgery between January 2020 to October 2024. Surgical volumes for emergency cases were not significantly altered by the COVID-19 pandemic during this period because this hospital was one of the main centers for surgery in the region. The study was approved by the institutional review board, and the requirement for informed consent was waived due to the retrospective nature of the analysis.

### Data collection

Data were abstracted from hospital records using a standardized data collection form. Variables collected included age, sex, surgeon type (NS vs GS), presentation delay (defined as > 12 h from symptom onset), procedure type (craniotomy, burr hole, elevation, craniectomy, shunt [primary and revision], or incision and drainage/washout), and early outcomes (postoperative complications within 30 days and length of hospital stay [LOS] in days). Follow-up was limited to 30 days because the majority of patients do not present for evaluation beyond this period. All records were reviewed for completeness and accuracy by the senior author (BHM), with discrepancies resolved by cross-referencing operative and discharge notes.

### Statistical analysis

Descriptive statistics were used to summarize patient characteristics, procedure types, and outcomes by provider group. Continuous variables were reported as medians with interquartile ranges [IQR], and categorical variables as counts and percentages. Group comparisons were conducted using the Mann–Whitney U test for continuous variables and Chi square with Yates’ correction test for categorical variables.

To account for differences in procedural complexity, a case-mix complexity score was developed for each procedure type, reflecting typical resource requirements and technical difficulty. Surgical procedures were stratified into two complexity tiers based on technical demands and resource requirements: tier 1 (routine) (score = 1) including elevation and washout/I&D procedures; tier 2 (complex) (score = 2) comprising shunt and craniotomy [11–13]. Craniotomies were considered complex because they were outside the scope of practice for the GS operating at this hospital and, for consistency of analysis, they were kept as complex even when an NS was added to the staff. Complexity-weighted case volumes were calculated for each provider type. Multivariable logistic regression was performed to evaluate the association between provider type and early postoperative complications, adjusting for age, sex, delayed presentation, and procedure type. Adjusted odds ratios (aOR) and 95% confidence intervals (CI) were reported. Statistical significance was defined as a two-sided p < 0.05. All analyses were performed using Python libraries.

### Outcomes

Primary outcome was the rate of early postoperative complications. Secondary outcomes included length of hospital stay, distribution of procedure types, delayed presentation rates, and complexity-weighted case volumes.

## Results

### Patient characteristics

**Fig. 1:**
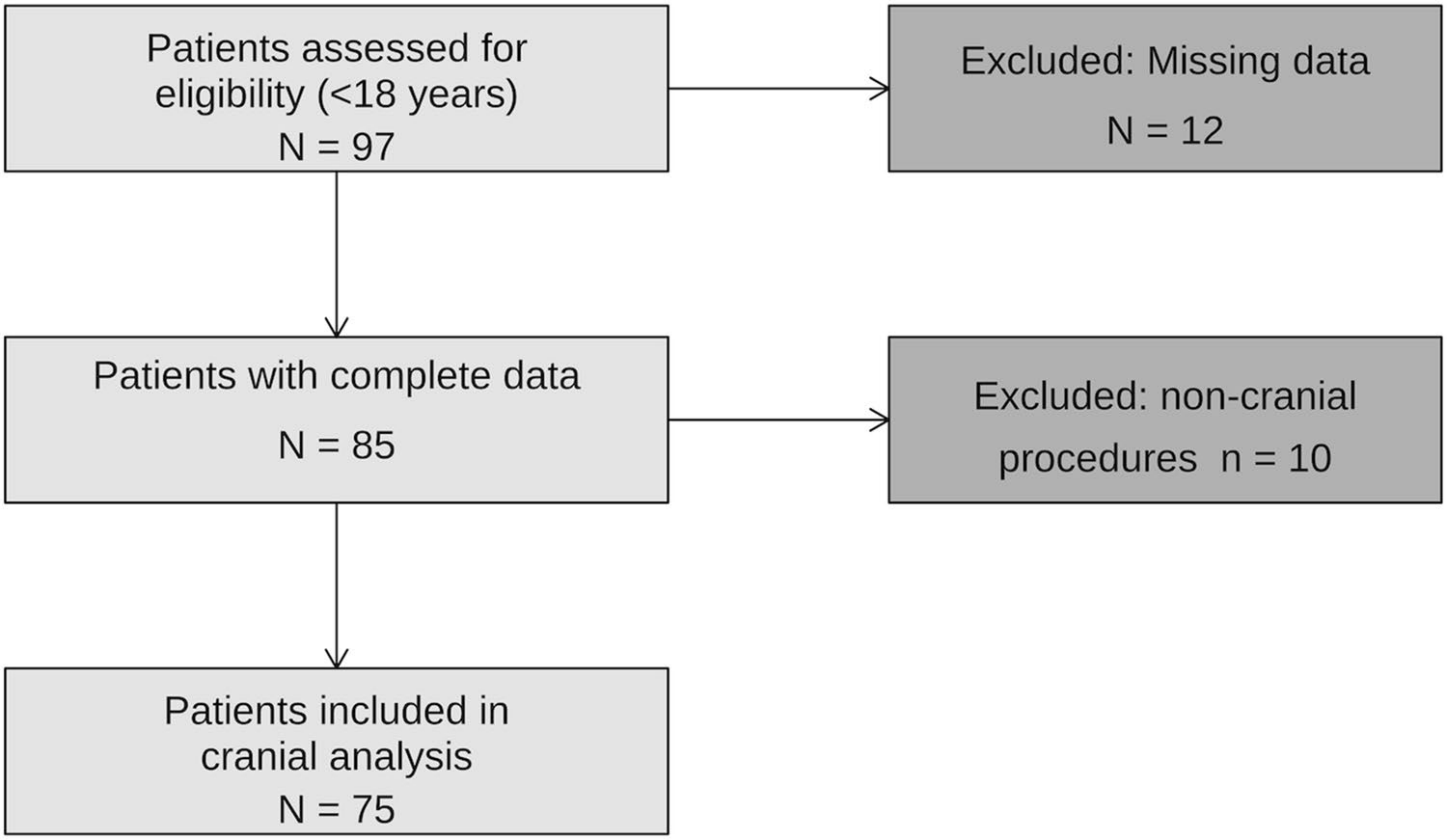
CONSORT flow diagram showing patient selection and exclusions

**Fig. 2:**
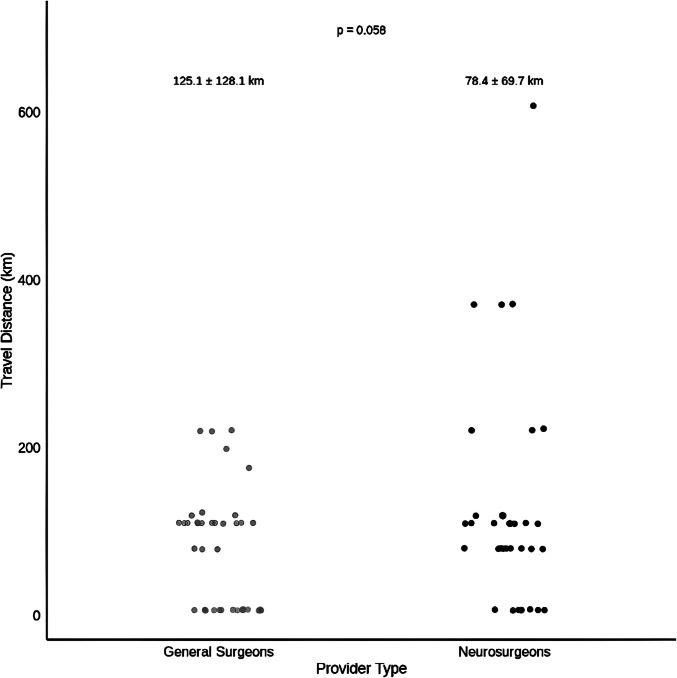
Distance to hospital by provider type (km)

Data were collected on 97 patients who were < 18 years old. Twelve patients were excluded for incomplete charts (Fig. 1). A total of 75 patients met inclusion criteria, of whom 36 (48.0%) underwent cranial procedures performed by NS and 39 (52.0%) by GS (Table 1). The mean travel distance was greater for patients treated by NS with patients traveling 125.1 ± 128.1 km versus 78.4 ± 69.7 km for GS (p = 0.058). This difference was not significant. (Fig. 2). The GS group was older (9.4 ± 5.3 vs 6.8 ± 5.8 years, p = 0.050). The majority of patients in both groups were male (83.3% NS vs 69.2% GS; p = 0.247). Delayed presentation, defined as arrival at the hospital more than 12 h after symptom onset, was similar in both groups (44.4% NS vs 41.0% GS patients; p = 0.948).

**Table 1:** Patient characteristics by provider type

Characteristic	NS	GS	Total	p-value
Number of patients, n	36	39	75	-
Age (years), mean ± SD	6.8 ± 5.8	9.4 ± 5.3	8.2 ± 5.7	0.050
Male, %	83.3	69.2	76.0	0.247
Delayed presentation, %	44.4	41.1	42.7	0.948
Travel distance (km), mean ± SD	125.1 ± 128.1	78.4 ± 69.7	100.8 ± 104.0	0.058

### Procedure types

The distribution of cranial procedures varied between provider groups. NS most frequently performed elevation (41.7%), followed by shunt (36.1%), washout/I&D (13.9%), and craniotomy (8.3%). GS most frequently performed elevation (56.4%), craniotomy (25.6%), washout/I&D (12.8%), and shunt (5.1%). Notably, shunt procedures were performed predominantly and more significantly by NS (36.1% vs 5.1%; p = 0.001), while craniotomies were more commonly performed by GS (25.6% vs 8.3%; p = 0.068) (Table 2).

**Table 2:** Distribution of procedures by provider type

Procedure	Surgeon	
	NS, n (%)	GS, n (%)
Elevation	15.0 (41.7)	22.0 (56.4)
Washout/I&D	5.0 (13.9)	5.0 (12.8)
Shunt	13.0 (36.1)	2.0 (5.1)
Craniotomy	3.0 (8.3)	10.0 (25.6)

### Outcomes

**Fig. 3:**
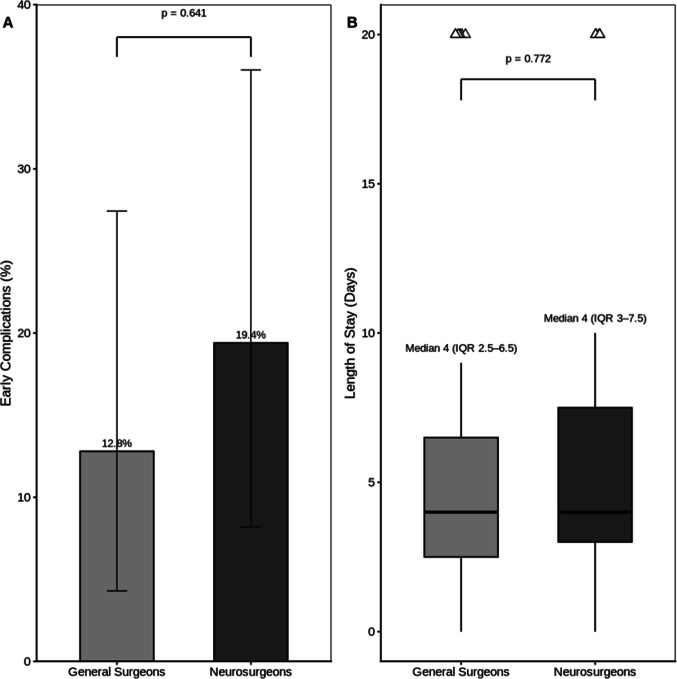
Outcomes by Provider. Panel A: Complication Rate by Provider (%). Panel B: Length of Stay by Provider (days)

Early postoperative complications occurred in 7 (19.4%) of NS cases and 5 (12.8%) of GS cases, with no statistically significant difference between providers (χ2 test with Yates’ correction, p = 0.641) as shown in Fig. 3A. To account for potential confounding factors, multivariable logistic regression analysis adjusting for age, sex, and delayed presentation was performed. After adjustment, there remained no statistically significant difference in the odds of complications between NS and GS (adjusted OR 1.25, 95% CI: 0.27–5.78; p = 0.773). The analysis also showed that there is no statistically significant difference in length of hospital stay between patients treated by GS and NS (p = 0.772); both groups have the same median LOS of 4 days, with slightly different interquartile ranges (NS: 4 [3–7.5] vs GS: 4 [2.5–6.5] days) as shown in Fig. 3B.

### Case-mix complexity analysis

**Fig. 4:**
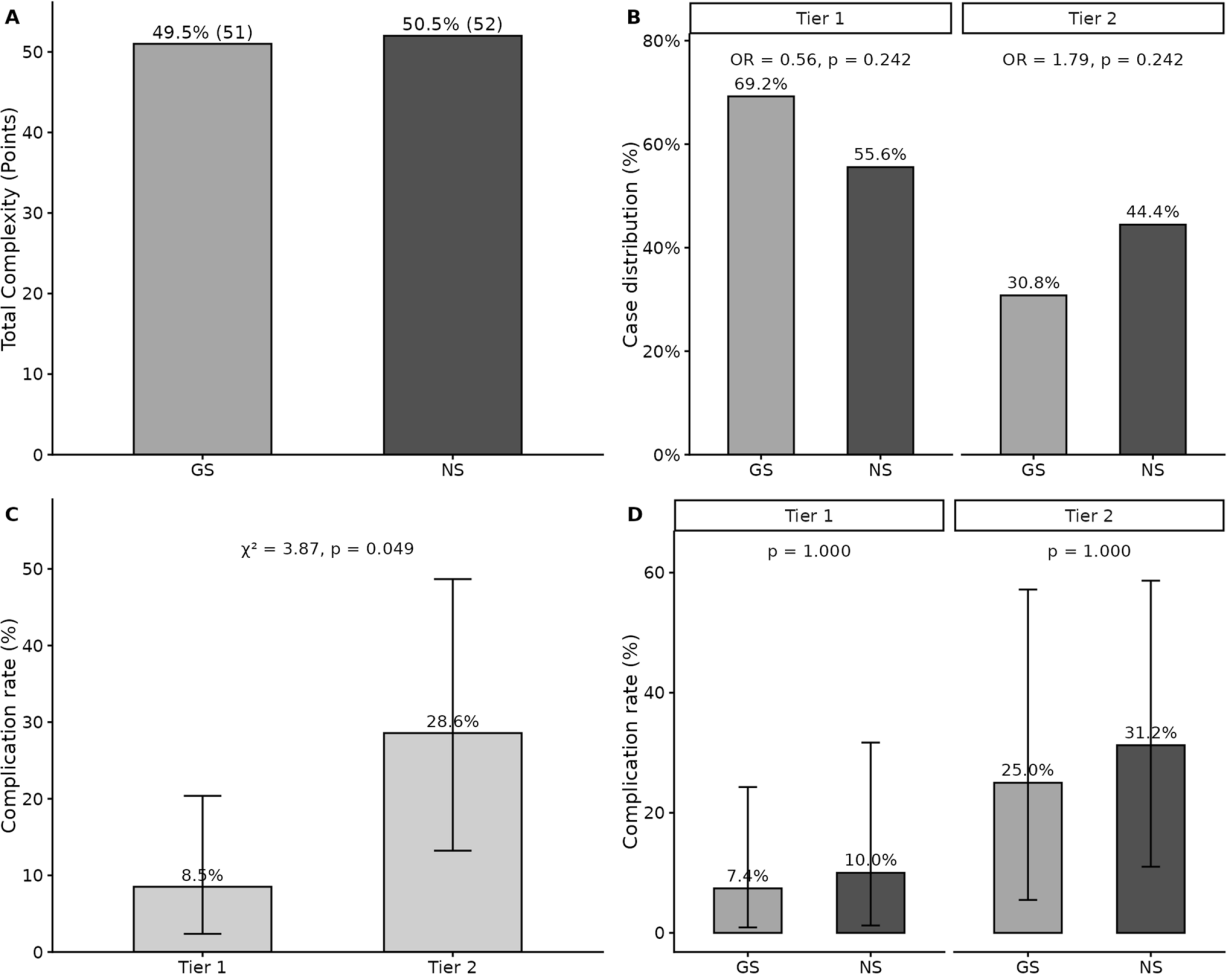
Complexity-weighted analysis of neurosurgical procedures by surgeon type. Panel A: Distribution of total complexity points between GS and NS. Panel B: Case distribution across complexity tiers by provider type with odds ratios and statistical comparisons. Panel C: Overall complication rates by complexity tier showing significant trend across tiers (p < 0.001). Panel D: Complication rates by complexity tier stratified by provider type with statistical comparisons

Case-mix analysis, focusing on complexity-weighted case volumes, procedure distribution, and complication rates using a two-tier system (routine [elevation and washout/I&D] tier 1 score = 1 vs complex [shunt and craniotomy] tier 2 score = 2) demonstrated that GS accounted for 49.5% of total complexity points (51 points), while NS contributed 50.5% (52 points) (Fig. 4A). The distribution of cases across tier 1 and tier 2 procedures did not differ significantly between the two groups (χ2 = 0.969, p = 0.325). GS performed 69.2% (27/39) of their cases as routine procedures, while NS performed 55.6% (20/36) as routine; conversely, NS performed a higher proportion of complex procedures (44.4%, 16/36) compared to GS (30.8%, 12/39), but these differences were not statistically significant (OR for routine: 1.79, p = 0.242; OR for complex: 0.56, p = 0.242) (Fig. 4B).

Complication rates for routine procedures (tier 1) were low in both groups (GS: 2/27, 7.4%; NS: 2/20, 10.0%), with no significant difference between providers (p = 1.000). For complex procedures (tier 2), complication rates were higher but again comparable (GS: 3/12, 25.0%; NS: 5/16, 31.2%; p = 1.000). The mean case complexity score was similar between providers (GS: 1.31 ± 0.47 vs NS: 1.44 ± 0.50; p = 0.228), indicating that overall case-mix complexity did not differ significantly. Although complication rates increased significantly with procedural complexity (χ2 = 3.89, p = 0.049 as shown in Fig. 4C, the differences in between provider types within either tier were not statistically significant (tier 1: p = 1.000; tier 2: p = 1.000) and complication rates remained comparable between surgeon types when stratified by complexity tier (Fig. 4D).

## Discussion

This analysis demonstrated no statistically or clinically significant differences in early postoperative outcomes including complications, reoperation, infection, or in-hospital mortality between GS and NS, even after multivariable adjustment for potential confounders such as age, sex, delay to presentation, and case complexity. These findings remained consistent when stratified by complexity tier, reinforcing the safety and viability of TS models in pediatric neurosurgery.

### Case complexity and outcomes

Although NS performed proportionally more shunts and GS managed more trauma-related cases, complexity-weighted volume was virtually identical (GS 49.5% vs NS 50.5%). Dewan et al. highlighted that LMIC systems cannot depend solely on pediatric NS to handle the expanding burden of complex procedures and advocated competency-based pathways for non-specialists [2]. Our findings that GS safely executed one-third of tier-2 cases (craniotomies and shunts) substantiates Dewan’s premise, demonstrating that competency-based up-skilling can extend the operative envelope of GS without compromising outcomes. Similarly, Asfaw et al. urged decentralization of complex pediatric cases within Ethiopia to avoid referral backlogs in Addis Ababa [5]. The data show that such decentralization is achievable when GSs receive targeted training and supervision.

### Role of travel distance and geography

**Fig. 5:**
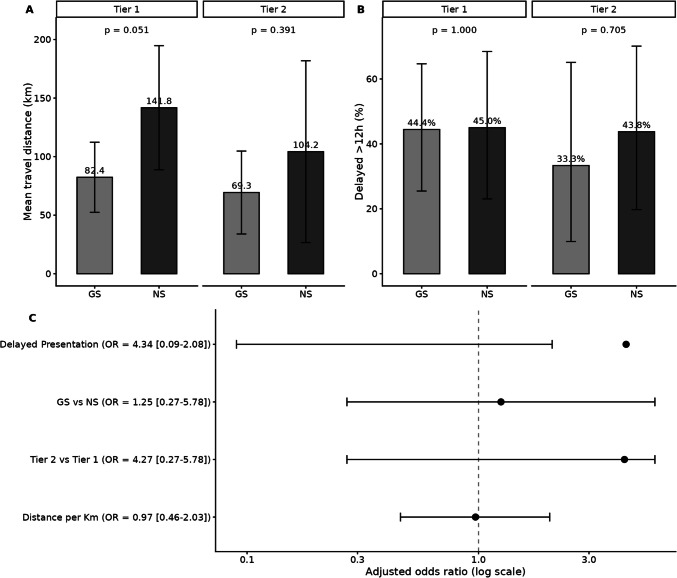
Drivers of Complications. Panel A: Mean travel distance for each complexity level, split by provider type. Panel B: Delay rates by provider and complexity. Panel C: Forest plot of adjusted odds ratios for complications

Travel distance was shorter for GS treated children but did not differ by complexity tier, suggesting that geographic proximity itself, not case severity, drives where patients present (Fig. 5). The Lancet Commission on High-Quality Health Systems stressed that long travel times erode the quality of time-sensitive care [6], while Shrime et al. demonstrated that every additional hour of travel increases both pre-operative mortality risk and catastrophic household expenditure [14]. Our results support those observations that by enabling GSs to manage emergency cranial cases closer to patients’ homes, the health system can mitigate prehospital deterioration and financial hardships, even when the procedures are complex.

### Alignment with global task-sharing literature

Adult neuro-trauma studies provide evidence for the effectiveness of supervised task-sharing. In the Philippines, Robertson et al. analyzed 650 emergency cranial cases and found no difference in 30-day mortality or complication rates when GS operated under NS oversight, attributing success to rigid post-operative pathways and compulsory NS consults [9]. In Tanzania, Ormond et al. reported that GS who completed dedicated neurosurgical rotations maintained low perioperative mortality once back at their district hospitals, underscoring the value of longitudinal mentorship [15]. At Mbarara Regional Referral Hospital in Uganda, Abdelgadir et al. documented GS-led emergency craniotomies with case-fatality rates comparable to international benchmarks after a tele-mentoring link with Kampala NS was established [16]. Our data extend these adult observations to the pediatric population. Although remote supervision has been reported elsewhere as a suitable task-sharing strategy, unstable internet connectivity in our setting precluded routine tele-mentoring; our findings therefore reflect an unsupervised GS model [17].

### Policy implications and national strategy

Ethiopia’s long-term plan to scale its neurosurgical workforce to 150 surgeons by 2035 is ambitious but likely insufficient given the pediatric population exceeds 59 million [5]. Our findings support expanding neurosurgical capacity through structured TS models that train GSs to perform both routine and select complex pediatric cranial procedures. This should be paired with a national framework that includes targeted competency-based training, telehealth-enabled supervision and robust outcome tracking and quality assurance. To ensure the safety and equity of this approach, rigorous outcome monitoring is essential. Establishing clear benchmarks and accountability mechanisms will help maintain quality standards and foster long-term trust in TS models of care. These findings are consistent with global surgical policy goals outlined in the Lancet Commission on Global Surgery and the World Health Organization’s Surgical Systems Strengthening framework [6, 18].

## Study limitations

This single-center retrospective study is subject to selection bias, documentation limitations, and may not fully represent other facilities. The complexity scoring system, while pragmatic, does not account for all operative nuances such as intraoperative blood loss and anesthetic risk. A further limitation of our study is that the available dataset did not allow for a more granular analysis of delay impact beyond a binary metric or further subclassification of craniotomy procedures (e.g., epidural vs. intradural), and therefore we used the broader category consistent with prior studies. Similarly, the terms ‘routine’ and ‘complex’ reflect previously published classification frameworks and were applied here for consistency, though we recognize that these definitions may vary by surgeon and clinical context. Finally, the small sample size may limit the power to detect modest differences in subgroup analyses.

## Future directions

Prospective multi‑center registries with standardized data are needed to validate these findings and evaluate cost‑effectiveness. Qualitative studies exploring caregiver satisfaction and surgeon perspectives will further refine TS models globally.

## Conclusion

In a high‑volume Ethiopian referral hospital, GS achieved early outcomes equivalent to NS for both routine and complex pediatric cranial operations. When embedded within a competency‑based, mentored framework, TS represents a pragmatic, immediately scalable bridge toward universal pediatric neurosurgical coverage.

## Data Availability

No datasets were generated or analysed during the current study.
